# Efficacy and safety of primary, early and late needle-knife fistulotomy for biliary access

**DOI:** 10.1038/s41598-021-96142-9

**Published:** 2021-08-17

**Authors:** Jorge Canena, Luís Lopes, João Fernandes, Gonçalo Alexandrino, Luísa Figueiredo, Marta Moreira, Tarcísio Araújo, Luís Lourenço, David Horta, Pietro Familiari, Mário Dinis-Ribeiro

**Affiliations:** 1 Department of Gastroenterology, Professor Doutor Fernando Fonseca Hospital, IC 19 PT–2720-276, Amadora, Portugal; 2 Department of Gastroenterology, Santa Luzia Hospital, Unidade Local de Saúde Alto Minho, Viana do Castelo, Portugal; 3 Department of Gastroenterology – Nova Medical School/Faculty of Medical Sciences, Lisbon, Portugal; 4 Department of Gastroenterology, Portuguese Oncology Institute of Porto, Porto, Portugal; 5 School of Medicine, Life and Health Sciences Research Institute (ICVS), University of Minho, Braga, Portugal; 6 ICVS/3B’s - PT Government Associate Laboratory, Braga/Guimarães, Portugal; 7 Cintesis – Center for Health Technology and Services Research, Porto, Portugal; 8 Digestive Endoscopy Unit, Agostino Gemelli University Hospital, Rome, Italy

**Keywords:** Gastroenterology, Medical research

## Abstract

European Society of Gastrointestinal Endoscopy recommends needle-knife fistulotomy (NKF) as the preferred precut technique. However, there is little information on whether NKF performed at different times is associated with different success and adverse event rates. We compared the outcomes of 3 different timings of NKF. This was an observational study conducted at 4 institutions and this was a retrospective analysis of prospectively collected data. We included 330 consecutive patients submitted to NKF attempt for biliary access. Patients were divided into three groups: NKF as an initial procedure for biliary access (group A, n = 121); early NKF defined as after 5 min, 5 attempts, or 2 pancreatic passages (group B, n = 99); and late NKF: after at least 10 min of unsuccessful standard biliary cannulation (group C, n = 110). We assessed the success rate of biliary cannulation at initial ERCP, time to perform NKF until biliary cannulation, overall biliary cannulation rate (second ERCP when initial failure), adverse event rate, and predictors of post-ERCP pancreatitis (PEP). The initial cannulation rate was 98%, 91% and 94% for groups A, B and C respectively, *p* = 0.08, whereas overall biliary cannulation rate was 100%, 95% and 98%, *p* = 0.115. The adverse event rate/PEP was 4.1%/2.5%, 7.1%/4% and 10.9%/8.2%, for groups A, B and C respectively, (*p* = 0.197 and *p* = 0.190). Median time for creating the fistula was A = 4.0 min, B = 3.2 min, and C = 5.6 min, *p* < 000.1. Each additional minute spent attempting cannulation increased the odds ratio (OR) for PEP by 1.072, and patients with 3 or more risk factors for pancreatitis had a higher chance of PEP. In conclusion, the timing of NFK does not appear to influence success rates but late NFK is associated with a higher time to create a fistula and an increased risk of pancreatitis. Primary NFK is associated with a high rate of success and a low rate of PEP and deserves additional investigation.

## Introduction

Endoscopic retrograde cholangiopancreatography (ERCP) is an advanced procedure that is widely used in the diagnosis and treatment of a variety of benign and malignant pancreatobiliary disorders^1–5^. Selective deep cannulation of the biliary tract is the most important prerequisite for successful therapeutic ERCP^6–8^. However, even experienced endoscopists may face technical problems when using standard techniques for biliary cannulation; therefore, selective access to biliary ducts mail fail in 5–35% of cases^6–9^. In this subset of patients, rescue techniques are needed to obtain deep cannulation of the desired duct. Precut techniques have emerged as a type of advanced technique that may be used as rescue techniques for accessing bile ducts. Recently, the European Society of Gastrointestinal Endoscopy (ESGE) has recommended needle-knife fistulotomy (NKF) as the preferred technique for precutting^10^. Furthermore, ESGE suggests that precutting should be used only by endoscopists who achieve selective biliary cannulation in more than 80% of cases using standard cannulation techniques^10^. However, there is no consensus about the timing of performing the NKF during an ERCP, especially because very few studies comparing NKF strategies are available. One study suggested that when comparing early NKF (after 5 min of ERCP) with late NKF (after 15 min of ERCP), although the overall cannulation rate was similar between the two groups, post-ERCP pancreatitis (PEP) and procedural duration were lower in the early NKF group^9^. One systematic review with meta-analysis investigated whether early precut biliary sphincterotomy was associated with a lower incidence of PEP when compared with repeated papillary cannulation attempts^11^. The abovementioned meta-analysis of randomized controlled trials (RCTs) showed a decreasing trend for PEP with early precut sphincterotomy but was not statistically significant. However, when the analysis was restricted to the two RCTs that employed fistulotomy, this technique significantly reduced the odds of PEP^11^. Furthermore, two recent studies have suggested that in a cohort of patients at high risk for PEP, primary NFK is feasible and safe, and when compared with patients submitted to standard cannulation, it is associated with significantly lower rates of PEP (0%) and higher rates of successful cannulation at initial ERCP^6,7^. In fact, at the moment, there is a clear lack of information on whether performing NKF at different timings is associated with different success rates and adverse event rates, mainly because this study has never been undertaken. Furthermore, when looking at the literature there are only 3 studies including patients submitted to primary NFK. Therefore, we conducted a study comparing the outcomes of three different timings of NKF for accessing the biliary system, namely, the initial method of cannulation, whether early (soon after a difficult cannulation was declared) or late (after multiple attempts with conventional techniques).

## Methods

### Patients and setting

This was an observational study conducted in four centers in which NKF is performed by two senior endoscopists. We conducted a retrospective analysis of prospectively collected data. Between January 2017 and February 2020, all of the consecutive patients who underwent NKF for biliary access in a naïve papilla were enrolled in the study and were followed prospectively. Patients with surgically altered anatomy or tumors of the papilla were excluded. Patients were divided into three groups: (a) NKF as an initial method for biliary access, (b) early NKF and (c) late NKF (see definitions below). During the study period patients submitted to NKF after the 6th minute and before the 10th minute, with more than 5 attempts and more than 2 pancreatic passages (endoscopist’s decision associated with the type of clinical situation during ERCP), were considered to be outside groups B and C and excluded from the analysis. The decision of the timing to perform NKF was left to the discretion of the endoscopist. In our institutions, the preferred rescue technique for difficult cannulation is NKF, which is undertaken, as far as timing is concerned, according to the preference of the endoscopist based on several issues, namely, the morphology of the ampulla of Vater or the position of the endoscope facing the major papilla, and trainee involvement. This procedure was performed preferentially in patients with a suitable morphology of the papilla, avoiding most of the times flat and diverticular papillae. Trainee involvement was associated, most of the times with late NKF and when a very poor position was achieved NKF was avoided. Since 2017, we began performing primary NKF initially in patients at high risk for PEP^6,7^ and later in 2018 in all patients for whom the endoscopist decided to perform the technique to lower the risk of PEP.

The data collected included patient demographics, indication for ERCP, underlying biliary pathology, therapeutic interventions, rate of success of NKF in the first ERCP or overall, after a second ERCP in initial failures, length of time for achieving biliary cannulation, length of time to create a successful fistula, intraprocedural adverse events and postprocedural adverse events (30-day follow-up). This study was conducted at 4 institutions (2 tertiary referral academic centers and 2 general district hospitals). The Ethics Committee at each institution approved this observational study (ULSAM ethics committee at Viana do Castelo Hospital, ULSBA Ethics Committee at Hospital de Beja, HPFA Ethics Committee at Amadora-Sintra Hospital and ULSNE Ethics Committee at Hospital Distrital de Mirandela). All research was performed in accordance with relevant guidelines/regulations, and informed consent was obtained from all participants and/or their legal guardians. This was an observational study without randomization (not a clinical trial) and therefore no clinical trial registration was undertaken.

### Outcomes and definitions

The primary outcomes were as follows: (1) initial cannulation rate for the three groups and (2) adverse event rate, with special attention paid to the PEP rate for the three groups. The secondary endpoints included median time to undertake the fistulotomy, median ERCP time until biliary cannulation at first ERCP, time to begin NKF, overall cannulation rate (after a second ERCP when there was failure of the first ERCP) and predictors of PEP.

NKF as the initial method was defined as using NKF as an initial procedure for biliary access without contact with the orifice of the papilla^6,7^. Early NFK was defined as the use of NKF after one or more of the following: more than 5 contacts with the papilla while attempting to cannulate; more than 5 min spent attempting to cannulate following visualization of the papilla or more than one unintended pancreatic duct cannulation or opacification, which is the recommendation of ESGE for defining difficult biliary cannulation^10^. Late NKF was defined as the use of NFK for biliary cannulation after at least 10 min of unsuccessful standard biliary cannulation attempts as suggested elsewhere^12–14^. The length of time for achieving biliary cannulation after starting cannulation attempts was defined as the length of time between the first contact with the ampulla of Vater and visualization of a guidewire in the biliary duct. The length of time for creating a fistula from the papilla to the biliary tract was defined as the length of time between the first contact of the needle-knife with the papilla and visualization of a guidewire in the biliary duct. The time to begin NKF was the length of time between the beginning of unsuccessful standard cannulation attempts and the first contact of the papilla with the needle-knife.

For the purpose of comparing the pancreatitis rate between groups, all patients were evaluated for the following patient-related risk factors for PEP as suggested elsewhere^6,7,15–17^: age 18–50, female, normal common bile duct (CBD) diameter (< 9 mm), normal serum bilirubin, previous acute pancreatitis and suspected sphincter of Oddi dysfunction (SOD). Post-ERCP complications, namely, PEP, ERCP bleeding and retroperitoneal perforation, were classified and graded according to consensus guidelines^7,18,19^.

### Intervention, endoscopists, and PEP prevention

We followed a standard protocol (supplementary material) that has been used in our institutions. ERCP procedures were performed in the prone position under sedation with propofol administration by an anesthesiologist. Although several ERCPists were involved in the ERCP procedures, as recommended elsewhere^20^, the only endoscopists performing NKF in the study (JC, LL) achieved selective biliary cannulation in more than 80% of the patients using standard access techniques. Furthermore, the abovementioned ERCPists had performed more than 8000 ERCPs in their careers with an annual load above 400 ERCPs/year and had performed more than 500 NKF in their careers and more than 30 NKF/year in the last 5 years. Other ERCPists involved in the study were ERCPists with more than one year of practice and at least 500 procedures accomplished. During the study period the trainees were endoscopists with limited experience in ERCP (less than one year of experience and less than 200 ERCPs performed/visualized). The trainees did not initiate the procedure in diverticular papillae. The trainees could undertake biliary cannulation attempts for at least 6 min and if deep cannulation was not obtained, a senior endoscopist took over. During the study period 4 trainees were involved in the procedures.

The details of the NFK technique have been extensively described by others and will not be addressed in this manuscript^6–9,20–26^. Prophylaxis of PEP was undertaken in all patients. Routine rectal administration of 100 mg of indomethacin or diclofenac immediately before ERCP was performed. In all cases of inadvertent guidewire passage into the pancreatic duct or pancreatic opacification, a prophylactic 5 Fr pancreatic stenting was placed as recommended elsewhere^10,12^.

### Statistical analysis

Qualitative variables are summarized using absolute and relative frequencies, and quantitative variables are summarized using the mean and standard deviation or the median and interquartile range, depending on their distribution profiles. The normality of the quantitative variables was assessed using the histogram distribution.

Relations between categorical variables were assessed using a chi-square test and Fisher´s exact test. Differences between two or more groups of independent non-normally distributed quantitative variables were evaluated using a Kruskal–Wallis test.

A sample of 326 patients will provide 80% power to detect an effect-size of 0.17 in pancreatitis rates between the three groups based on a chi-square test, assuming a pancreatitis rate of 1% among primary fistulotomy^6,7^, and a one-tailed alpha of 0.05.

To explain the risk of pancreatitis, a binomial logistic regression model with the following 6 predictors was performed: 2 interval predictors: (1) age and (2) time attempting cannulation (minutes) and 4 indicator predictors: (3) sex (*male, as base case*) and (4) number of risk factors for pancreatitis (*0 risk factors, as base case*), (5) malignant biliary stenosis (*no stenosis, as base case*) and (6) common bile duct stones (*no stones, as base case*).

The null hypothesis was rejected when the test statistics *p* values were less than < 0.05. Statistical analysis, sample size calculation and graphics were performed using Stata software (StataCorp. 2015. Stata Statistical Software: Release 14. College Station, TX: StataCorp LP).

## Results

### Patients

Between January 2017 and February 2020, 330 patients (147 males and 183 females) with a mean age of 74.1 years (range 18–97 years) fulfilled group selection criteria and were enrolled in the study. The three groups defined earlier in the methods section were as follows: group A (primary NFK, n = 121), group B (early NFK, n = 99), and group C (late NFK, n = 110). During the study period additional existing 7 patients were excluded from the analysis because NKF was undertaken outside the strict criteria for inclusion in group B and before the 10th minute to be considered for inclusion in group C. Patients of groups B and C resulted from a group of patients in which NKF was used as a rescue technique in 11% of the cases. There were no additional patients excluded. Included patient demographics, number of PEP patient-related risk factors, cholangiographic findings and placement of pancreatic stents are summarized in Table 1. There were no significant differences in sex, PEP risk factors, or pancreatic stent placement among the three groups. However significant differences were found for age and several cholangiographic findings namely CBD stones and malignant biliary strictures between groups A, B and C.

**Table 1:** Patient demographics, patient-related factors for PEP, use of pancreatic stents and cholangiographic findings for the 3 NFK groups.

	Primary NKF n (%)	Early NKF n (%)	Late NKF n (%)	*p*
Age (years), mean (range)	71.3 (18–97)	75.6 (18–96)	74.9 (30–96)	0.018**
Male sex	59 (48.7%)	42 (42.4%)	46 (41.8%)	0.698
PEP risk factors, mean (SD)	1.428 (0.966)	1.200 (0.807)	1.409 (0.951)	0.287
**PEP risk factors**				
0	18 (14.9%)	20 (20.2%)	18 (16.4%)	0.522
1	49 (40.5%)	43 (43.4%)	45 (40.9%)	
2	39 (32.2%)	32 (32.3%)	33 (30.0%)	
3	11 (9.1%)	4 (4.0%)	12 (10.9%)	
4	4 (3.3%)	0	2 (1.8%)	
5	0	0	0	
6	0	0	0	
**Cholangiographic findings**				
CBD stones	106 (87.6%)	58 (58.6%)	56 (50.9%)	< 0.001***
Malignant biliary stricture	10 (8.3%)	23 (23.2%)	34 (30.9%)	< 0.001***
Leaks	2 (1.7%)	4 (4.0%)	4 (3.6%)	0.475
Other findings	3 (1.7%)	14 (14.1%)	16 (14.5%)	< 0.001***
Pancreatic stents	0	5 (5.1%)	3 (2.7%)	0.262

**p < 0.05.

***p < 0.01.

### Primary outcomes

Primary outcomes are shown in Table 2. Overall, regarding the initial cannulation rate, selective biliary cannulation was obtained in 311/330 (94.2%) of the patients. In group A, initial success was obtained in 118/121 (97.5%) of the patients. Following the initial NFK attempt, biliary cannulation was successful in 90/99 (90.9%) patients in group B and in 103/110 (93.6%) patients in group C. There were no significant differences in the initial cannulation rate of the 3 groups (*P* = 0.083).

**Table 2:** Primary and secondary outcomes: cannulation rate, adverse events rate and length of time to create a fistula and time to achieve biliary cannulation in the 3 groups.

	Primary NKF n (%)	Early NKF n (%)	Late NKF n (%)	*p*
**Biliary cannulation**				
Success in first ERCP	118/121 (97.5%)	90/99 (90.9%)	103/110 (93.6%)	0.083
Overall biliary cannulation	121 (100%)	94 (94.9%)	108 (98.2%)	0.115
**Adverse events**				
Overall	5 (4.1%)	7 (7.1%)	12 (10.9%)	0.197
Pancreatitis	3 (2.5%)	4 (4.0%)	9 (8.2%)	0.190
Bleeding	2 (21.7%)	3 (3.0%)	3 (2.7%)	0.750
Time to begin NKF, Median (p25-75)	–	3.6 (1.8–4.0)	10.3 (10.0–16.7)	< 0.001***
NKF duration, Median (p25-75)	4.0 (4.1–7.2)	3.2 (2.5–4.5)	5.6 (3.5–10.5)	< 0.001***
Time to cannulation,Median (p25-75)	4.0 (4.01–7.1)	8.1 (6.6–9.1)	18.5 (13.2–26.8)	< 0.001***

p25-p75: lower and upper limit of the interquartile range.

***p < 0.01.

In total, adverse events occurred in 24/330 (7.3%) of the patients, being most frequent in group C (10.9%), and these differences were not statistically significant among the 3 groups (*P* = 0.197). In total, pancreatitis occurred in 16/330 (4.8%) of the patients, being most frequent in group C (8.2%), and the differences among the 3 groups was not statistically significant (*P* = 0.190). When comparing differences between pair of groups, statistically significance was not achieved: group A versus group B (*p* = 0.649), group A versus group C (0.092) and group B versus group C (0.240). All PEP cases were mild and resolved with conservative management. Bleeding occurred in 2 patients in group A, 3 patients in group B and 3 patients in group C. All cases of bleeding occurred after ERCP (3, less than 24 h after ERCP; 2, on day 1 after ERCP; and 3, more than 48 h after ERCP). In five patients, the bleeding was mild, and no additional measures were undertaken. In 3 patients (one in each group), one additional ERCP was performed to control the bleeding, and transfusion was needed (2 units for each patient). These patients resumed anticoagulant therapy on the day after ERCP. No patients developed perforation or cholangitis after ERCP.

### Secondary outcomes

**Figure 1:**
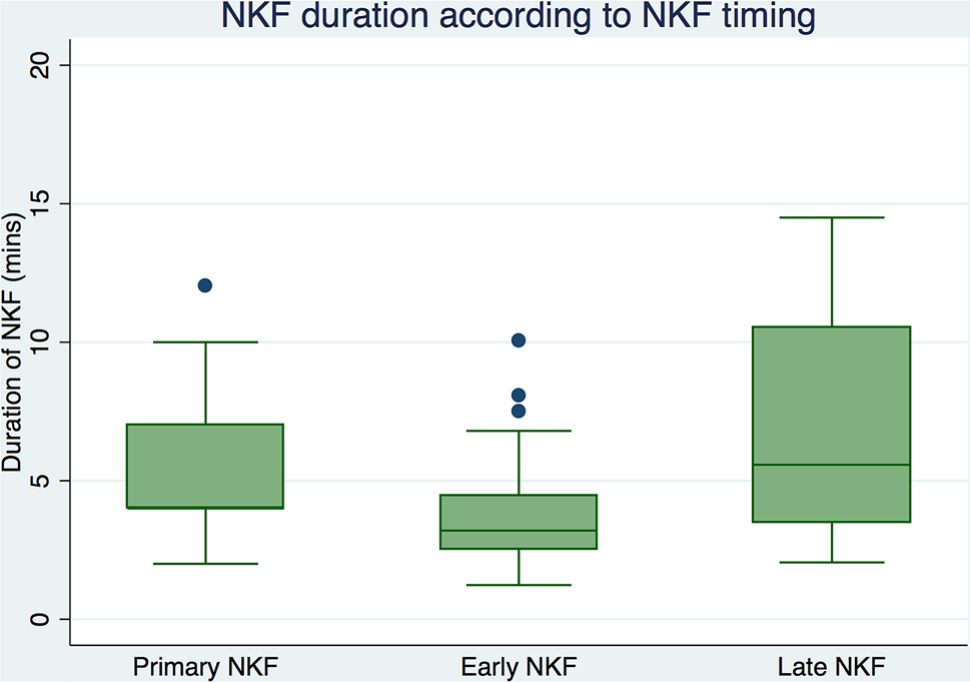
Statistically different times for performing needle-knife fistulotomy (NKF) with success among the 3 groups.

In total, 323/330 (97.9%) of the patients had successful biliary cannulation after the second ERCP using NFK (Table 2). From the first to the second ERCP, there was an increase in successful biliary access from 94.2% to 97.9%, representing an increase of 12/330 (3.6%) of the patients. The second ERCP was performed after a median time of 6.2 days (range: 4–9 days). Of the remaining 7 patients, 3 had a successful biliary cannulation using other advanced techniques, and 4 did not undergo a second ERCP. Selective biliary cannulation was more effective in group A than in groups B and C, but there were no significant differences in the overall cannulation rates of the 3 groups (*P* = 0.115). When comparing the length of time for performing a successful fistulotomy for the 3 groups, patients in group C had a significant longer time to complete the fistula and achieve deep biliary cannulation (*P* < 0.001) [A vs. B (*P* = 0.275); A vs. C (*P* < 0.001); B vs C (*P* < 0.001)] (Fig. 1). The total median cannulation time was significantly longer in group C than in groups B and A (18.45 vs. 8.07 vs. 4.02 min, respectively) (*P* < 0.001), [A vs. B (*P* < 0.001); A vs. C (*P* < 0.001); B vs C (*P* < 0.001)].

### Regression model for evaluating predictors of pancreatitis

To assess potential predictors of pancreatitis, a univariate analysis was performed to assess potential associations (Table 3), followed by a logistic regression with six predictors (Table 4). The results from this model demonstrated that the marginal and separate effect of time to cannulation was 1.072, which means that each additional minute attempting cannulation increased the odds ratio for PEP by 1.072, holding all other predictors constant.

**Table 3:** Univariate analysis for evaluating possible associations for pancreatitis.

	Pancreatitis		*p*
	No, n (%)	Yes, n (%)	
Age	74.5 (61.6–81.9)	64.9 (59.7–79.4)	0.2314
Sex			1.000
Female	172 (93.7%)	11 (6.3%)	
Male	138 (93.7%)	9 (6.3%)	
CBD stones			0.496
No	139 (93.3%)	10 (6.7%)	
Yes	172 (94.9%)	9 (5.1%)	
Malignant biliary stricture			0.678
No	248 (93.9%)	16 (6.1%)	
Yes	63 (95.4%)	3 (4.6%)	
PEP risk factors			0.004
0	55 (98.2%)	1 (1.8%)	
1	132 (96.4%)	5 (3.6%)	
2	100 (96.2%)	4 (3.8%)	
3	24 (88.9%)	3 (11.1%)	
4	3 (50.0%)	3 (50.0%)	
NKF timing			0.175
Primary	118 (97.5%)	3 (2.5%)	
Early	95 (96.0%)	4 (4.0%)	
Late	101 (92.8%)	9 (8.2%)	
Cannulation time, median (p25; p75)	7.58 (4.00–13.91)	14.91 (8.04–25.02)	0.0034

p25–p75: lower and upper limit of the interquartile range.

**Table 4:** Logistic regression to evaluate predictors of pancreatitis.

Variables	Odds Ratio	Robust SE	95% CI		*p*
Age	1.017	0.020	0.979	1.057	0.370
**Sex**					
Female	0.4013	0.31	0.0922	1.853	0.249
**CBD stones**					
Yes	1.249	0.957	0.278	5.606	0.771
**Malignant biliary stricture**					
Yes	0.657	0.618	0.103	4.159	0.656
Time to cannulation (min)	1.072	0.025	1.024	1.123	0.003*
**No. of PEP risk factors**					
1	3.340	3.492	0.455	25.423	0.232
2	3.675	4.527	0.328	41.108	0.290
3	24.116	12.768	1.540	377.436	0.023*
4	106.869	33.002	5.015	2276.988	0.003*

SE: standard error; CI: confidence interval.

*p < 0.05.

Regarding the number of PEP risk factors, 3 or more risk factors increased the odds of developing pancreatitis. A patient with 3 risk factors had an odds ratio of 24.116 when compared with a patient with no risk factor for PEP. Interestingly, when a patient had 2 or fewer risk factors, the risk of developing PEP was not different from that of a patient with no risk factors for PEP.

## Discussion

According to our findings in this observational multicenter study comparing a large cohort of patients submitted to 3 different timings of NKF (primary, early and late), we did not observe different success rates in biliary cannulation between the 3 groups.

Although the rate of adverse events is lower in primary NKF and higher in late NKF, these differences did not achieve statistical significance, which could result from the 20% chance of not discovering differences despite their existence (using 80% power in the sample size estimation) or may result from an underestimation of the risk of pancreatitis in the primary NKF group and/or overestimation of the rate of adverse events between NKF timings.

However, late NKF was associated with a significantly longer time to complete the fistula and a trend toward a higher rate of pancreatitis resulting from the prolonged number of contacts with the papilla (length of time for achieving biliary cannulation). In the regression model, each additional minute attempting cannulation increased the odds ratio (OR) for PEP by 1.072, and patients with 3 or more risk factors for pancreatitis had a higher chance of PEP. Finally, primary NKF was feasible, with a 97.5% initial success rate and a low rate of pancreatitis (2.5%).

Since 2001 NKF is the preferred rescue technique in our units. Our group works in 4 hospitals in which the same protocol has been implemented. In these 4 units NKF is performed by the two coordinators (JC, LL) which have been using this advanced technique for more than 20 years.

Another issue is which patients are selected to primary, early or late NKF? In our units the preferred method is conventional cannulation using the guidewire assisted technique. NKF is the preferred rescue technique and most of the times we preferred to undertake it in an early strategy trying to avoid that a prolonged ERCP time will increase the risk of adverse events. However, there are several factors that have led us to extend the time of conventional cannulation beyond the 10 min barrier namely when trainees are involved, intra-diverticular papillae, small papillae or other disadvantageous types of morphologies including tumoral infiltration that makes the performance of rescue advanced techniques more difficult. Interestingly, in the current study, the time to complete the fistula was similar in groups A and B, but there was a statistically significant difference of a longer time to complete the fistula in group C, suggesting that multiple attempts of standard cannulation may create trauma and edema at the ampulla of Vater and may therefore increase the time to achieve success when completing the fistula but not the success rate itself (see below).

Although some of the pioneers of ERCP attribute its conception and use to Claude Liguory in Paris, during the decade of 70 of the XX century, its original description in the literature is attributed in 1978 to an Italian endoscopist from Bologna, named Caletti^27^. In 2016, the European Society of Gastrointestinal Endoscopy (ESGE) recommended needle-knife fistulotomy as the preferred technique for precutting mainly because the NKF technique significantly reduced the odds of PEP^10^.

The popularity of precut techniques (conventional and fistulotomy) was low in the past because old studies reported a high rate of complications with precut techniques, namely, perforations, bleeding, and pancreatitis^28–30^. However, there is now robust evidence that the use of NKF is safe and successful in the hands of experienced endoscopists^6–8,25,26^. Three large series are available including from 204 to 352 patients and a technical success ranging from 81 to 92%^8,25,26^ (Table 5). These studies were done without the use of indomethacin and NKF was used after a difficult cannulation in a late strategy. In the current study, we undertook NKF in a large cohort of 330 patients (the second largest reported in the literature), and we observed an initial success rate in 311/330 (94.2%) of the patients, which compared with the 2 large series from the USA^25^ and Ireland^26^. Furthermore, as suggested in other reports^8,9^ our initial cannulation success was further extended with the same technique in a second ERCP (from 94.2 to 97.9%).

**Table 5:** Case series evaluating outcomes of needle-knife fistulotomy.

References	Patients (n)	Technical success (%)	Pancreatitis rate (%)	Pancreatitis prophylaxis	Time to undertake NFK (minutes)
Harewood and Baron^25^	253	92	11	No	Late (after 20 min)
Donnellan et al.^26^	352	90.1	0.3	No	Late (no time reported)
Lopes et al.^8^	204	81	6.4	No	Late (after 12–15 min)

Primary NKF has gained interest in recent years. In 2016, a group from South Korea reported the utility of NKF as an initial method of biliary access in a group of patients with one or more patient-related risk factors for PEP, which they considered to be a population at a high risk of PEP^6^. They enrolled 55 patients and observed a 96.3% CBD cannulation success rate and a 0% rate of pancreatitis without using indomethacin for PEP prevention. Four years later, the same group conducted an RCT comparing 87 patients submitted to the conventional cannulation method versus 96 patients allocated to primary NFK, and all of the patients were considered to be at high risk for pancreatitis^7^. The investigators observed a significant difference in technical success and complications between the two groups in favor of the NFK group. Again, they reported a 0% rate of pancreatitis in the NKF group, and none of the patients in either group were submitted to PEP prophylaxis with NSAIDs. In an editorial related to this research, some questions were raised^31^, the primary question being if the nonuse of NSAIDs in the conventional group posed an artificial advantage in favor of the NFK group. This was a clear caveat, and with a large probability, the use of prophylactic measures for PEP in the conventional group was likely to blunt the differences or even negate the statistical significance of the pancreatitis rate in favor of the NFK group. Furthermore, regarding the abovementioned findings, the pancreatitis rate of 0% was impressive and deserved further research. However, what was not written in either paper (the RCT or the editorial) or in the original paper of 2016 is the fact that in 2008, a group from Iran first reported primary fistulotomy as a cannulation method^32^. Furthermore, they performed it in an RCT comparing a population with a mix of patients (with low and high risk for pancreatitis). In this RCT, the authors performed exactly the same study of the Korean group comparing primary NKF (106 patients) vs. the standard cannulation method (112 patients). Furthermore, the RCT was conducted in 2003/2004 without PEP prophylaxis. The researchers in Iran observed no significant differences between groups with regard to successful cannulation and pancreatitis. In the NKF group, pancreatitis was observed in 1.9% of the patients vs. 2.6% of the patients in the standard cannulation group.

There is a clear rationale behind the use of primary NKF. First, PEP is believed to occur, in part, due to trauma and edema during cannulation attempts causing transient obstruction of the pancreatic duct. Furthermore, after cannulation, sphincterotomy will cause thermal injury of the shared orifice, which will further contribute to the blockage of the pancreatic duct. Therefore, the use of NKF is theoretically a protector from pancreatitis risk because the incision made by the NK is made above and to the left of the biliary orifice, avoiding contact with and thermal injury to the pancreatic duct. Another issue is whether NKF is a rescue procedure with a high success rate in experienced hands and why it is not used as a first biliary method of cannulation, especially in high-risk patients. However, NKF is a blind process, and the position of the CBD may not always be located at the usual position in the infundibulum; therefore, theoretically, in a minority of patients, thermal injury of the pancreatic duct may occur, and pancreatitis may develop. Therefore, a systematic 0% pancreatitis rate in patients submitted to primary NKF deserves further research. For example, in the RCT conducted in Korea, 3.1% of the patients had contrast injection of the pancreatic duct, and in 8.3% of the patients, selective pancreatic cannulation was achieved^7^. Furthermore, no indomethacin was used, only one pancreatic stent was placed in the NKF group, and a 0% pancreatitis rate was still obtained. In the current study, 121 patients underwent NKF with a pancreatitis rate of 2.5% (3/121) and a success rate at initial ERCP of 97.5%. This success rate was similar to the 2 studies conducted by the Korean group (96.3% and 97.9%), but the pancreatitis rate was not 0%, and our rate was similar to the pancreatitis rate observed in the study from Iran (1.9%)^32^. A possible explanation can be obtained in our regression model in which patients with 3 or more risk factors are at an increased risk of pancreatitis. In our population of 121 patients submitted to NKF, we observed 3 cases of mild pancreatitis in 3 patients with four risk factors. In these 3 patients, all fistulas were created in less than 4 min, and no pancreatic cannulation was seen. Therefore, a larger series of patients submitted to primary NKF is needed, including patients with low and high risk and those with different endoscopic appearances of the ampulla of Vater. Further, more comparisons of primary NFK with standard cannulation methods are needed, especially if both groups are submitted to PEP prophylaxis. Which patients we select to primary NKF? In our units we started undertaking primary NKF in patients with several risk factors for pancreatitis and in large papillae with a long intramural segment as suggested by others. However, with experience (personal data, in press) we have extended primary fistulotomy to patients with almost all types of papillae with several risk factors, co-morbidities or when a fast ERCP procedure was mandatory.

In the current study, when comparing the adverse event rate in the three groups, there was a trend toward a higher rate of complications from group A to B and from group B to C, and this trend was caused by the increasing number of pancreatitis events from group A to B (2.5 vs. 4.0%) and from group B to C (4.0% vs. 8.2%). This increasing rate of pancreatitis (almost doubling the rate from group A to B and from B to C) was in line with the significant differences in the time to achieve biliary cannulation among the three groups, and this difference was due to the time of 18.5 min observed in group C. Taken together, these findings suggest that the late NKF strategy was associated with a prolonged time to achieve biliary cannulation and that the trend toward a higher rate of pancreatitis in group C developed as a consequence of the duration of the ERCP, which was linked to the increased duration of the attempts to cannulate the bile duct and not to the NKF. Our regression model clearly shows that each additional minute spent attempting cannulation increases the odds ratio for PEP by 1.072. Also, off interest in our univariate and multivariate analysis significant differences between groups A, B and C found in Table 1 proved to be of no significance for the PEP model. Further The fact that early classic precut was associated with a trend toward a lower incidence of PEP than that after late precut has been suggested by a recent systematic review and meta-analysis evaluating 7 RCTs and 7 non-RCTs^33^. However, 12 of the 14 studies included the conventional precut, and only two included patients underwent fistulotomy^32,33^. In these 2 abovementioned studies, the end point was to compare early fistulotomy to standard cannulation and not to late fistulotomy. Only one study had a significant pancreatitis rate in favor of early NKF when compared with the numerous-attempt strategy^33^. However, in this study, randomization was made only after 10 min of standard attempts, and after the initial 10 min, the endoscopist was free to extend the procedural time as long as he wanted. Therefore, the authors concluded that pancreatitis develops because of the time spent on cannulation attempts.

In the current study, the three timings of NKF were not associated with different success rates of biliary cannulation, and this fact has not been clearly shown in previous studies because most of them compare patients submitted to early NKF with patients submitted to standard cannulation, and in this group, some of the patients are eventually submitted to a late NKF; therefore, the number of patients who undertake a late fistulotomy is small, and most of the time, these numbers are not easy to access in the studies. In one of the abovementioned RCTs, the success rate between early and late NKF was similar. The success of early NFK was observed in 63/77 (81.8%) of the patients and in 42/50 (84%) of the patients submitted to late NKF^33^. In the study from Iran, success was obtained in 83% of the patients who underwent primary NKF and in 16/20 (80%) of the patients who underwent late NKF^32^^.^ Therefore, from these small numbers, we could suspect what our study showed for the first time that late NKF may be associated with a prolonged time to complete the fistula, but the success rate is not different from that with other NFK timings. Of interest in our study and in both studies from Korea, the success rate associated with primary NKF is very high, and it is not clear if these observations result from expertise or from adequate selection of the papilla morphology, or if it is easier to undertake an NKF in an untouched papilla, or if the findings are a consequence of all factors combined. Again, more research in this emerging area is needed.

The current study has several limitations: although patients were prospectively enrolled, patient selection bias exists because it is not a randomized and controlled study. Therefore, the result obtained accordingly does not have a high level of evidence. Furthermore, NFK was performed by experienced endoscopists with a special interest in NKF, and thus, the results may not be representative of the average NFK outcomes in community hospitals. The strength of our study lies in the large number of patients and in the design of the study. To the best of our knowledge, this is the first study to compare three strategies of NKF and is the second largest NKF series ever reported. In addition, our study is only the 4th paper in the literature to include patients with primary NKF and of those papers is the one with the largest number of enrolled patients.

In conclusion and based on the limitations of the study we suggest that the timing of NKF does not appear to influence biliary access success rates when comparing three different timings. Late NFK as a consequence of the time expended in cannulation attempts is associated with a significantly longer time to complete the fistula, which is probably related to trauma of the ampulla of Vater during prolonged attempts to cannulate the biliary tract. Moreover, late NFK is associated with a trend toward a higher rate of adverse events (including pancreatitis). In the regression model, each additional minute spent attempting cannulation increases the odds ratio (OR) for PEP by 1.072, and patients with 3 or more risk factors for pancreatitis have a higher chance of contracting PEP. Altogether, there is a clear suggestion that prolonged duration of the ERCP and therefore the undertaking of late NKF should be avoided. Primary NKF when feasible is associated with high rates of success and a low rate of complications and therefore is a new, promising alternative deserving further research. Finally, NKF, was shown in a large series to be a safe procedure, with a low rate of complications and a high success rate, and this success can be further amplified with a second ERCP using the same technique.

## Supplementary Information

Supplementary Information.

## References

[1] Canena J, Liberato M, Coutinho AP, Marques I, Romão C, Veiga PM, Neves BC (2014). Predictive value of cholangioscopy after endoscopic management of postcholecystectomy bile duct strictures with an increasing number of plastic stents: A prospective study. Gastrointest. Endosc..

[2] Canena J, Coimbra J, Carvalho D, Rodrigues C, Silva M, Costa M, Horta D, Mateus-Dias A, Seves I, Ramos G, Coutinho AP, Romão C, Veiga PM (2014). Endoscopic bilio-duodenal bypass: Outcomes of primary and revision efficacy of combined metallic stents in malignant duodenal and biliary obstruction. Dig. Dis. Sci..

[3] Canena J, Liberato M, Meireles L, Marques I, Romão C, Coutinho AP, Neves BC, Veiga PM (2015). A Non-randomized study in consecutive patients with postcholecystectomy refractory biliary leaks submitted to endoscopic management using multiple plastic stents or fully covered self-expandable metal stents. Gastrointest. Endosc..

[4] Canena J (2018). Once upon a time a guideline was used for the evaluation of suspected choledocholithiasis: A fairy tale or a nightmare?. GE Port. J. Gastroenterol..

[5] Canena J, Lopes L, Fernandes J, Alexandrino G, Lourenço L, Libânio D, Horta D, Giestas S, Reis J (2019). Outcomes of single-operator cholangioscopy-guided lithotripsy in patients with difficult biliary and pancreatic stones. GE Port. J. Gastroenterol..

[6] Jin YH, Jeong S, Lee DH (2016). Utility of needle-knife fistulotomy as an initial method of biliary cannulation to prevent post-ERCP pancreatitis in a highly selected at-risk group: a single-arm prospective feasibility study. Gastrointest. Endosc..

[7] Jang S, Kim D, Cho J, Jeong S, Park JS, Lee DH, Kwon CI, Koh DH, Park SW, Lee TH, Lee HS (2020). Primary needle-knife fistulotomy versus conventional cannulation method in a high-risk cohort of post-endoscopic retrograde cholangiopancreatography pancreatitis. Am. J. Gastroenterol..

[8] Lopes L, Dinis-Ribeiro M, Rolanda C (2014). Safety and efficacy of precut needle-knife fistulotomy. Scand. J. Gastroenterol.

[9] Lopes L, Dinis-Ribeiro M, Rolanda C (2014). Early precut fistulotomy for biliary access: Time to change the paradigm of “the later, the better”?. Gastrointest. Endosc..

[10] Testoni P, Mariana A, Aabakken L, Arvanitakis M, Bories E, Costamagna G, Devière J, Dinis-Ribeiro M, Dumonceau JM, Giovannini M, Gyokeres T, Hafner M, Halttunen J, Hassan C, Lopes L, Papanikolaou IS, Tham TC, Tringali A, van Hooft J, Williams EJ (2016). Papillary cannulation and sphincterotomy techniques at ERCP: European Society of Gastrointestinal Endoscopy (ESGE) Clinical Guideline. Endoscopy.

[11] Choudhary A, Winn J, Siddique S, Arif M, Arif Z, Hammoud G, Puli SR, Ibdah JA, Bechtold ML (2014). Effect of precut sphincterotomy on post-endoscopic retrograde cholangio-pancreatography pancreatitis: A systematic review and meta-analysis. World. J. Gastroenterol..

[12] Dumonceau JM, Andriulli A, Elmunzer BJ, Mariani A, Meister T, Deviere J, Marek T, Baron TH, Hassan C, Testoni P, Kapral C (2014). Prophylaxis of post-ERCP pancreatitis: European Society of Gastrointestinal Endoscopy (ESGE) guideline—Updated June 2014. Endoscopy.

[13] Dumonceau J-M, Rigaux J, Kahaleh M, Gomez CM, Vandermeeren A, Devière J (2010). Prophylaxis of post-ERCP pancreatitis: a practice survey. Gastrointest. Endosc..

[14] Chandrasekhara V, Khashab MA, Muthusamy R, Acosta RD, Agrawal D, Bruining DH, Eloubeidi MA, Fanelli RD, Faulx AL, Gurudu SR, Kothari S, Lightdale JR, Qumseya BJ, Shaukat A, Wang A, Wani SB, Yang J, DeWitt JM (2017). Adverse events associated with ERCP-ASGE guideline. Gastrointest. Endosc..

[15] Elmunzer BJ, Scheiman JM, Lehman GA, Chak A, Mosler P, Higgins PD, Hayward RA, Romagnuolo J, Elta GH, Sherman S, Waljee AK, Repaka A, Atkinson MR, Cote GA, Kwon RS, McHenry L, Piraka CR, Wamsteker EJ, Watkins JL, Korsnes SJ, Schmidt SE, Turner SM, Nicholson S, Fogel EL, Cooperative for Outcomes Research in Endoscopy (USCORE) (2012). A randomized trial of rectal indomethacin to prevent post-ERCP pancreatitis. N. Engl. J. Med..

[16] Freeman ML, DiSario JA, Nelson DB, Fennerty MB, Lee JG, Bjorkman DJ, Overby CS, Aas J, Ryan ME, Bochna GS, Shaw MJ, Snady HW, Erickson RV, Moore JP, Roel JP (2001). Risk factors for post-ERCP pancreatitis: A prospective, multicenter study. Gastrointest. Endosc..

[17] Wang P, Li ZS, Liu F, Ren X, Lu NH, Fan ZN, Huang Q, Zhang X, He LP, Sun WS, Zhao Q, Shi RH, Tian ZB, Li YQ, Li W, Zhi FC (2009). Risk factors for ERCP-related complications: A prospective multicenter study. Am. J. Gastroenterol..

[18] Cotton PB, Eisen GM, Aabakken L, Baron TH, Hutter MM, Jacobson BC, Mergener K, Nemcek A, Petersen BT, Petrini JL, Pike IM, Rabeneck L, Romagnuolo J, Vargo JJ (2010). A lexicon for endoscopic adverse events: Report of an ASGE workshop. Gastrointest. Endosc..

[19] Cotton PB, Lehman G, Vennes J, Geenen JE, Russell RC, Meyers WC, Liguory C, Nickl N (1991). Endoscopic sphincterotomy complications and their management: An attempt at consensus. Gastrointest. Endosc..

[20] Mavrogiannis C, Liatsos C, Romanos A, Petoumenos C, Nakos A, Karvountzis G (1999). Needle-knife fistulotomy versus needle-knife papillotomy for the treatment of common bile duct stones. Gastrointest. Endosc..

[21] Katsinelos P, Gkagkalis S, Chatzimavroudis G, Beltsis A, Terzoudis S, Zavos C, Gatopoulou A, Lazaraki G, Vasiliadis T, Kountouras J (2012). Comparison of three types of precut technique to achieve common bile duct cannulation: A retrospective analysis of 274 cases. Dig. Dis. Sci..

[22] Abu-Hamda EM, Baron TH, Simmons DT, Petersen BT (2005). A retrospective comparison of outcomes using three different precut needle knife techniques for biliary cannulation. J. Clin. Gastroenterol..

[23] Horiuchi A, Nakayama Y, Kajiyama M, Tanaka N (2007). Effect of precut sphincterotomy on biliary cannulation based on the characteristics of the major duodenal papilla. Clin. Gastroenterol. Hepatol..

[24] Lee TH, Bang BW, Park SH, Jeong S, Lee DH, Kim SJ (2011). Precut fistulotomy for difficult biliary cannulation: Is it a risky preference in relation to the experience of an endoscopist?. Dig Dis. Sci..

[25] Harewood GC, Baron TH (2002). As assessment of the learning curve for precut biliary sphincterotomy. Am. J. Gastroenterol..

[26] Donnellan F, Zeb F, Courtney G, Aftab AR (2010). Suprapapillary needle-knife fistulotomy: A safe and effective method for accessing the biliary system. Surg. Endosc..

[27] Calleti GC, Vandelli A, Bolondi L, Fontana G, Labò G (1978). Endoscopic Retrograde Cholangiography (ERC) through artifitial endoscopic choledocho-duodenal fistula. Endoscopy.

[28] Kasmin FE, Cohen D, Batra S, Cohen SA, Siegel JH (1996). Needle-knife sphincterotomy in a tertiary referral center: Efficacy and complications. Gastrointest. Endosc..

[29] Rabenstein T, Ruppert T, Schneider HT, Hahn EG, Ell C (1997). Benefits and risks of needle-knife papillotomy. Gastrointest. Endosc..

[30] Freeman ML, Nelson DB, Sherman S, Haber GB, Herman ME, Dorsher PJ, Moore JP, Fennerty MB, Ryan ME, Shaw MJ, Lande JD, Pheley AM (1996). Complications of endoscopic biliary sphincterotomy. N. Engl. J. Med..

[31] Chen D, Keswani R (2020). Is needle knife fistulotomy a shortcut to preventing postendoscopic retrograde pancreatitis?. Am. J. Gastroenterol..

[32] Khatibian M, Sotoudehmanesh R, Ali-Asgari A, Movahedi Z, Kolahdoozan S (2008). Needle-Knife fistulotomy versus standard method for cannulation of common boile ducy: A randomized controlled trial. Arch. Iranian Med..

[33] Manes G, Di Giorgio PD, Repici A, Macarri G, Ardizzone S, Porro GB (2009). An analysis of the factors associated with the development of complications in patients undergoing precut sphincterotomy: A prospective. Controlled randomized, multicenter study. Am. J. Gastroenterol..

