# Supplementary material for: Efficacy and safety of primary, early and late needle-knife fistulotomy for biliary access
Source: Sci Rep. 2021 Aug 17;11:16658. doi: 10.1038/s41598-021-96142-9 (PMC8371017; doi:10.1038/s41598-021-96142-9)
Supplement: Supplementary file 1 — Supplementary Information. [file 41598_2021_96142_MOESM1_ESM.pdf]

ERCP FORM

Process number ULSAM: ______________________

**1 - ERCP HOSPITAL ID:**		**2 - ERCP DATA** (DD-MM-YY)**:**	
**3 - SNS NUMBER:**		**4 - BIRTHDAY** (DD-MM-YY)**:**	
**5 - SEX:**	Male ❑1 Female ❑2	**6 - NATIONALITY:**	Portuguese ❑1 Other ❑2

**7 - CENTER**
A ❑1 B ❑2 C ❑3 D ❑4
**8 - ENDOSCOPIST**
LL ❑1 JC ❑2
**9 - TRAINEE BEGAN THE ERCP?**
No ❑1 Yes ❑2

**10 - PATIENT ORIGIN**	**11 - SETTING**
From the hospital ❑1 Referred from other hospital ❑2	Inpatient ❑1 Outpatient ❑2

**12 - ERCP INDICATION** (FROM ASGE PROPER INDICATIONS)	
The jaundiced patient suspected of having biliary obstruction	❑1
The patient without jaundiced whose clinical and biochemical or imaging data suggest pancreatic duct or biliary tract disease	❑2
Evaluation of signs or symptoms suggesting pancreatic malignancy when results of direct imaging (eg, EUS, US, Computed tomography (CT), magnetic resonance imaging (MRI) are equivocal or normal	❑3
Evaluation of pancreatitis of unknown etiology	❑4
Preoperative evaluation of the patient with chronic pancreatitis and/or pseudocyst	❑5
Evaluation of the sphincter of Oddi by manometry	❑6
Endoscopic sphincterotomy for choledocholithiasis	❑7
Endoscopic sphincterotomy for papillary stenosis or sphincter of Oddi dysfunction	❑8
Endoscopic sphincterotomy to facilitate placement of biliary stents or dila n of biliary strictures	❑9
Endoscopic sphincterotomy for sump syndrome	❑10
Endoscopic sphincterotomy for choledochocele involving the major papilla	❑11
Endoscopic sphincterotomy for ampullary carcinoma in pa ents who are not candidates for surgery	❑12
Endoscopic sphincterotomy to facilitate access to the pancrea c duct	❑13
Stent placement across benign strictures	❑14
Stent placement across malignant strictures	❑15
Stent placement across fistulae or postoperative bile leak	❑16
Stent placement in high-risk patients with large unremovable common duct stones	❑17
Dilation of ductal strictures	❑18
Nasobiliary drain placement	❑19
Pancreatic pseudocyst drainage in appropriate cases	❑20
Balloon dilation of the papilla	❑21
Tissue sampling from pancreatic of bile ducts	❑22
Ampullectomy of adenomatous neoplasms of the major papilla	❑23
Therapy of disorders of the biliary and pacreatic ducts	❑24
Facilitation of cholangioscopy and/or pancreatoscopy	❑25

ERCP FORM

13 - LABORATORY TESTS BEFORE ERCP (24 hours prior to ERCP)	
1 - Hemoglobin (g/dl)	
2 - Platelets (10^3/uL)	
3 - Total bilirubin (mg/dl)	
4 - Direct bilirubin (mg/dl)	
5 - AST (U/l)	
6 - ALT (U/l)	
7 - AP (U/l)	
8 - G-GT (U/l)	
9 - Amilase (U/l)	
10 - INR	
11 - CA 19.9 (U/ml)	
12 - CEA (U/ml)	

14 - DILATION IN IMAGING STUDIES	
1 - Cholecystectomy	No ❑1 Yes ❑2

15 - ERCP PROPHYLAXIS			
No ❑1	100 mg diclofenac (retal) ❑2	100 mg indometacin (retal) ❑3	Pancreatic stent ❑4

16 - ERCP TIMER (hh:mm:ss)	
17.1 - Scope entering the mouth	
17.2 - Start biliary cannulation attempts (trainee)	
17.3 - Start biliary cannulation attempts (expert)	
39 - Start NKF	
43 - Biliary attempt	
17.4 - Deep biliary cannulation achieved	
17.5 - Scope exiting the mouth	

17 - VIANA CLASSIFICATION						
Type I ❑1	Type IIa ❑2	Type IIb ❑3	Type IIc ❑4	Type IIIa ❑5	Type IIIb ❑6	Type IV ❑7

18 - ORAL PORTION OF THE MAJOR PAPILLA	
18.1 - MEASUREMENT BY THE BIOPSY FORCEPS	
1 - Longitudinal diameter (mm)	
2 - Transversal diameter (mm)	

19 - BILIARY CANNULATION DIFFICULTY DIMENSIONS ASSESSMENT		
1 - I can position the duodenoscope in the short positioning	No ❑1	Yes ❑2
2 - I can get the papilla in the desire position for biliary cannulation*	No ❑1	Yes ❑2
3 - Facing the papilla in the desire position for biliary cannulation, i can move the duodenoscope to the position that i want in D2	No ❑1	Yes ❑2
4 - The tip of the duodenoscope responds in a on-to-one mode to the movements in the handle/wheels of the scope	No ❑1	Yes ❑2
5 - The duodenal arc seems to be fixed	No ❑1	Yes ❑2

*The axis of the CBD is aligned with the axis of the catheter

ERCP FORM

20 - BILIARY CANNULATION TECHNIQUE
Standard ☐$_1$ NKF ☐$_2$ Classic precut ☐$_3$ Double-wire technique ☐$_4$ Transpancreatic sphincterotomy ☐$_5$ Other ☐$_6$

21 - PANCREATIC STENT FOR POST-ERCP PANCREATITIS PROPHYLAXIS
No ☐$_1$ Before biliary cannulation attempts ☐$_2$ During biliary cannulation attempts ☐$_3$ After biliary cannulation attempts ☐$_4$

22 - PANCREATIC CANNULATION	
1 - Pancreatic cannulation	No ☐$_1$ Yes ☐$_2$
2 - Number of passages with the wire/catheter	Number ______ NA ☐$_0$
3 - Contrast injected	NA ☐$_0$ No ☐$_1$ Yes ☐$_2$
4 - Pancreatic acinarization	NA ☐$_0$ No ☐$_1$ Yes ☐$_2$
5 - Pancreatic stent for PEP inserted?	NA ☐$_0$ No ☐$_1$ Yes ☐$_2$
6 - Stend diameter (Fr)	Size ________ NA ☐$_0$
7 - Stent lenght (cm)	Size ________ NA ☐$_0$

23 - TECHNIQUES PERFORMED	
1 - Sphincterotomy	No ☐$_1$ Yes ☐$_2$
2 - Stent insertion	No ☐$_1$ Yes ☐$_2$
3 - Balloon sweepage	No ☐$_1$ Yes ☐$_2$
4 - Basket sweepage	No ☐$_1$ Yes ☐$_2$
5 - Balloon sphincteroplasty	No ☐$_1$ Yes ☐$_2$
6 - Balloon dilation after sphincterotomy	No ☐$_1$ Yes ☐$_2$
7 - Biliary cytology	No ☐$_1$ Yes ☐$_2$
8 - Spyglass	No ☐$_1$ Yes ☐$_2$
9 - Stone extraction	No ☐$_1$ Yes ☐$_2$
10 - Other ____________________	No ☐$_1$ Yes ☐$_2$

24 - CHOLANGIOGRAPHIC FIDINGS	
1 - Normal	No ☐$_1$ Yes ☐$_2$
2 - Stones	No ☐$_1$ Yes ☐$_2$
3 - Stenosis	No ☐$_1$ Yes ☐$_2$
4 - Leaks	No ☐$_1$ Yes ☐$_2$
5 - Non specific biliary dilation	No ☐$_1$ Yes ☐$_2$
6 - Other ________________	No ☐$_1$ Yes ☐$_2$

25 - ADVERSE EVENTS				
	27.1 - During the procedure	27.2 - Day after	27.3 - After 30 days	27.4 - Severity grading
1 - Pancreatitis*	NA ☐$_0$	No ☐$_1$ Yes ☐$_2$	No ☐$_1$ Yes ☐$_2$	NA ☐$_0$ Mild ☐$_1$ Moderate ☐$_2$ Yes ☐$_3$
2 - Bleeding**	No ☐$_1$ Yes ☐$_2$	No ☐$_1$ Yes ☐$_2$	No ☐$_1$ Yes ☐$_2$	NA ☐$_0$ Mild ☐$_1$ Moderate ☐$_2$ Yes ☐$_3$
3 - Perforation***	No ☐$_1$ Yes ☐$_2$	No ☐$_1$ Yes ☐$_2$	No ☐$_1$ Yes ☐$_2$	NA ☐$_0$ Mild ☐$_1$ Moderate ☐$_2$ Yes ☐$_3$
4 - Cholangitis	NA ☐$_0$	No ☐$_1$ Yes ☐$_2$	No ☐$_1$ Yes ☐$_2$	NA ☐$_0$
6 - Other	No ☐$_1$ Yes ☐$_2$	No ☐$_1$ Yes ☐$_2$	No ☐$_1$ Yes ☐$_2$	NA ☐$_0$

*Severity of pancreatitis: mild - clinical pancreatitis, amylase >3 times normal at least 24h after ERCP, requiring admission up to 3 days; moderate - hospitalization of 4-10 days; severe - hospitalization for > 10 days or hemorrhagic pancreatitis necrosis, pseudocyst or percutaneous/surgical intervention

**Severity of bleeding: mild - clinical evidence of bleeding decrease in hemoglobin to <3 g/L and no need for transfusion; moderate - transfusion (≤4 units) and no angiographic intervention or surgery; transfusion (≥5 units) or intervention

***Severity of perforation: mild - possible or only very slight leak of fluid or contrast, treatable by fluids and suction for ≤3 days; moderate - any definite perforation treated for 4-10 days; severe - medical treatment for > 10 days or intervention (percutaneous or surgical

ERCP FORM

26 - CBD MEASUREMENTS (MM)		
	MEASUREMENTS CBD	MEASUREMENTS ENDOSCOPE
3 - 1 cm above the papilla		

27 - PHOTOGRAPH?	
No ☐1	Yes ☐2

28 - OBSERVATIONS

29 - PRIMARY NKF?	No ☐1 Yes ☐2

30 - SUCCEFULL BILIARY CANNULATION IN THE FIRST ERCP?	No ☐1 Yes ☐2

31 - OVERALL BILIARY CANNULATION?	No ☐1 Yes ☐2

32 - SECOND ERCP DATE:	

33 - ODDI DISFUNCTION:	

34 - PREVIOUS PANCREATITIS:	No ☐0 Yes ☐1
